# Salmonella Meningitis and Systemic Lupus Erythematosus: A Case Report

**DOI:** 10.7759/cureus.77577

**Published:** 2025-01-17

**Authors:** Khalid Agrad, Aziza Kantri, Hicham Bakkali, Chafik El Kettani

**Affiliations:** 1 Anesthesiology and Critical Care, Mohammed VI International University Hospital, Mohammed VI University of Health Sciences, Casablanca, MAR; 2 Anesthesiology and Critical Care, Cheikh Khalifa International University Hospital, Mohammed VI University of Health Sciences, Casablanca, MAR; 3 Anesthesiology and Critical Care, Military Training Hospital Mohammed V, Mohammed V University, Rabat, MAR

**Keywords:** antibiotic therapy, immunosuppressive drug, meningitis, salmonella, systemic lupus erythematosus

## Abstract

Salmonella meningitis is an uncommon infection in adults. We report the case of a 44-year-old woman with a history of lupus on immunosuppressive treatment with mycophenolate mofetil and prednisolone who presented with confusional syndrome preceded by headache and fever. A lumbar puncture revealed bacterial meningitis with Salmonella species. She was treated with ceftriaxone with a good clinical and biological outcome.

## Introduction

Salmonella are gram-negative bacilli with more than 2,600 known serotypes. They are mainly responsible for foodborne gastroenteritis, typhoid, and paratyphoid fevers. They are thought to be responsible for 31% of foodborne deaths in the United States [1].

Salmonella meningitis remains rare and often affects neonates and young children, particularly in developing countries. In adults, this condition is exceptional, and only a few cases have been described, mainly in patients with immunodepression [2].

In this article, we report the case of a patient treated with immunosuppressants who was diagnosed with Salmonella meningitis.

## Case presentation

This case of a 44-year-old woman with a history of systemic lupus erythematosus (SLE) was complicated by renal involvement (class V + III lupus glomerulonephritis) with normal renal function. She was being treated with prednisolone (70 mg/day) in combination with hydroxychloroquine sulfate and had been started on mycophenolate mofetil (Cellcept° 2 g/day) 10 days before admission. She also had arterial hypertension treated with a triple combination (perindopril, indapamide, and amlodipine).

She presented to the emergency department with a fever of three days' duration, associated with a headache that had worsened since the morning of admission, with the onset of confusion. Her vital signs on admission were as follows: temperature of 40°C, blood pressure of 130/80 mm Hg, heart rate of 120 beats/min, respiratory rate of 16 breaths/min, and O_2_ saturation of 100% on room air. She was confused (Glasgow Coma Score of 13) with no sensorimotor deficits. Examination revealed no neck stiffness or rash.

A cerebral CT scan without contrast was performed and showed no abnormalities. A biological work-up was performed and showed the following as in Table 1.

**Table 1:** Laboratory findings in the patient

Test	Normal values	Day 1	Day 3	Day 5	Day 8
Blood leukocyte count, 10^3^/mm^3^	4-11	14.22	7.16	11.06	11.51
Lymphocyte (%)	-	6.3	5.6	11.5	16.8
Lymphocyte count, 10^3^/mm^3^	1- 4.3	0.9	0.4	1.27	1.93
Neutrophil (%)	-	88.7	91.6	84.2	76.4
Neutrophil count, 10^3^/mm^3^	1.4-7.7	12.6	6.6	9.3	8.8
Monocyte (%)	-	5	2.8	4.2	6.3
Monocyte count, 10^3^/mm^3^	0.18-1	0.71	0.2	0.46	0.73
Basophil (%)	-	0.00	0.00	0.01	0.02
Basophil count, 10^3^/mm^3^	<0.11	0.00	0.00	0.01	0.02
Erythrocyte count, 10^12^/L	4.28-6	3.83	4.09	4.11	4.16
Hemoglobin, g/dL	13-18	12.2	12.9	12.9	13
Hematocrit (%)	39-53	36.1	39	39.2	39
Platelet count, 10^3^/mm	150-400	243	266	310	439
C-reactive protein level, mg/L	<6	251	53.3	23.1	5.4
Procalcitonine, ng/ml	<0.5	0.80	0.45	-	0.30
Creatinine, mg/L	6-12	9.1	-	-	7.2
Blood urea nitrogen, g/L	0.15-0.45	0.51	-	-	0.55

The hemogram showed hyperleukocytosis of 14.22 × 10³ cells/mm³ with a predominance of neutrophils (88.7%), hemoglobin of 12.2 g/dL, and platelets of 243 × 10³ cells/mm³. Additionally, there was a marked biological inflammatory syndrome with a CRP of 251 mg/L and a slightly positive procalcitonin of 0.80 ng/ml.

A lumbar puncture was performed, which revealed a cloudy fluid with a high protein concentration of 2.88 g/L and a collapsed cerebrospinal fluid glucose level of 0.05 g/L on chemical examination. The CSF cell count showed the presence of 450 WBC/ mm^3^, including 30% neutrophils and 70% lymphocytes, with direct examination and Gram staining revealing the presence of a gram-negative coccobacillus (Table 2).

**Table 2:** Lumbar puncture results CSF: cerebrospinal fluid, WBC: white blood cells

CSF study	Reference range	Results
Appearance	Claire	Cloudy
Glucose level (g/L)	O.4-0.7	<0.05
Protein level (g/L)	0.15-0.45	2.88
WBC count (cells /mm^3^)	<5	450
Neutrophil (%)	-	30
Lymphocytes (%)	-	70
Gram stain	Negative	Gram-negative cocobacillus
Bacterial culture	Negative	Salmonella Species
Viral panel	Negative	Negative

Blood cultures were also taken and came back positive for the same germ. The patient was admitted to our intensive care unit, where she was started on antibiotic therapy with ceftriaxone 2 g/12 hours, corticosteroid therapy was continued, and mycophenolate mofetil (Cellcept°) was suspended. CSF and blood cultures were positive on the third day of admission, with isolation of Salmonella in CSF and blood. An antibiotic susceptibility test showed that the species was susceptible to ceftriaxone and that treatment should be continued for three weeks.

The patient experienced a favorable outcome, achieving a return to normal consciousness following three days of antibiotic treatment. After four days in intensive care, the patient was transferred to inpatient care. HIV serology and tests for hepatitis B and C were all negative. Additionally, a complement fraction assay was within normal limits, and protein electrophoresis and immunoglobulin levels were also normal, effectively ruling out an underlying immunodeficiency state. The case of Salmonella meningitis was attributed to immunodeficiency resulting from long-term corticosteroid therapy and treatment with mycophenolate mofetil.

The patient was discharged from the hospital after eight days, and treatment with ceftriaxone will continue at home for another two weeks.

## Discussion

Since the first case of Salmonella meningitis was described by Gohn in 1907 [3], this disease has remained rare, accounting for only 0.8% to 6% of all cases of bacterial meningitis, although there are variations between countries: its incidence is estimated at 0.1% in the UK but 12% in Malawi [4]. It mainly affects children under the age of five years, especially babies (6%) and newborns (16%) [5-7].

In adults, Salmonella meningitis is rare; it mainly affects patients with lymphoma, acute leukemia, sickle cell anemia, or AIDS, and patients on immunosuppressive drugs, in whom the prognosis is poor [8-10]. In the literature, most cases of Salmonella meningitis involve patients with HIV; salmonella infections are common in this population, especially in developing countries [11].

Patients with SLE are at increased risk of developing various infections. This susceptibility is due to various cellular and humoral immune deficiencies: complement deficiency, cytokine abnormalities, the presence of autoantibodies, dysfunction of neutrophils, lymphocytes, and the reticuloendothelial system, as well as immunosuppressive and cytotoxic drugs, all of which have been reported to play an important role in the increased risk of infections [10,12]. Salmonella infections are more common in these patients. Specific risk factors reported in the literature include corticosteroids and other immunosuppressive drugs, renal insufficiency, hemolytic anemia, and ineffective granulocyte and monocyte phagocytosis [13-15]. However, meningeal infection remains rare in this type of patient. In a series of 12 SLE patients, 11 of whom were women, Gerona and Navarra described joint involvement in five patients and meningeal involvement in a single 26-year-old female patient treated with corticosteroids [16].

Vargas reported a case of Salmonella meningitis in a series of 22 SLE patients with central nervous system infection [10]. In this case, the patient had several risk factors: SLE on immunosuppressive treatment with a combination of corticosteroids and mycophenolate mofetil, as well as hydroxychloroquine.

Another problem that can be caused by invasive salmonella infections is the emergence of antibiotic resistance. In a series of 662 non-typhi Salmonella isolates, 438 isolates (66.2%) were resistant to at least one antimicrobial agent. In 211 cases, the isolates were resistant to three or more antibiotics. The prevalence of resistance to ampicillin was 41.1%, and to sulfamethoxazole was 50.4%. Few isolates were resistant to cephalosporins, especially cefotaxime (4.1%) and cefepime (2.7%). Ciprofloxacin was ineffective in 5.6% of cases [17]. One publication even reported a case of colistin resistance in an HIV-positive patient with Salmonella meningitis [18]. These observations sound the alarm about the potential difficulties we may face in the future in treating these infections.

In our patient, the species isolated was intermediate to ampicillin but sensitive to ceftriaxone. We started her on ceftriaxone (2 g/12 hours), which resulted in a good clinical and biological outcome, although a follow-up LP was not performed. She was discharged after eight days in the hospital, and her treatment was continued for 21 days with good follow-up. It should be noted that a complementary immunodeficiency test was performed and showed no other predisposing factors, in particular a negative HIV serology.

## Conclusions

It is believed that this is the first case of Salmonella meningitis in a patient followed and treated for SLE reported in our country. This case involves a 44-year-old woman with SLE and lupus nephritis on immunosuppressive therapy who developed Salmonella meningitis. She presented with fever, headache, and confusion and was diagnosed through CSF analysis and culture. Prompt initiation of ceftriaxone and suspension of mycophenolate mofetil led to rapid clinical improvement. The infection was attributed to immunosuppression from corticosteroids and mycophenolate. The patient was discharged after eight days with continued antibiotic therapy at home, highlighting the importance of early diagnosis and management of infections in immunocompromised patients.
